# Autophagic Vacuolation Induced by Excess ROS Generation in HABP1/p32/gC1qR Overexpressing Fibroblasts and Its Reversal by Polymeric Hyaluronan 

**DOI:** 10.1371/journal.pone.0078131

**Published:** 2013-10-18

**Authors:** Paramita Saha, Anindya Roy Chowdhury, Shubhra Dutta, Soumya Chatterjee, Ilora Ghosh, Kasturi Datta

**Affiliations:** Biochemistry and Toxicology Laboratory, School of Environmental Sciences, Jawaharlal Nehru University, New Delhi, India; Rajiv Gandhi Centre for Biotechnology, India

## Abstract

The ubiquitous hyaladherin, hyaluronan-binding protein 1 (HABP1/p32/gC1qR) upon stable overexpression in normal fibroblasts (F-HABP07) has been reported to induce mitochondrial dysfunction, growth retardation and apoptosis after 72 h of growth. HABP1 has been observed to accumulate in the mitochondria resulting in generation of excess Reactive Oxygen Species (ROS), mitochondrial Ca^++^ efflux and drop in mitochondrial membrane potential. In the present study, autophagic vacuolation was detected with monodansylcadaverin (MDC) staining from 36 h to 60 h of culture period along with elevated level of ROS in F-HABP07 cells. Increased expression of autophagic markers like MAP-LC3-II, Beclin 1 and autophagic modulator, DRAM confirmed the occurrence of the phenomenon. Reduced vacuole formation was observed upon treatment with 3-MA, a known PI3 kinase inhibitor, only at 32 h and was ineffective if treated later, as high ROS level was already attained. Treatment of F111 and F-HABP07 cells with bafilomycin A1 further indicated an increase in autophagosome formation along with autophagic degradation in HABP1 overexpressed fibroblasts. Comparison between normal fibroblast (F111) and F-HABP07 cells indicate reduced level of polymeric HA, its depolymerization and perturbed HA-HABP1 interaction in F-HABP07. Interestingly, supplementation of polymeric HA, an endogenous ROS scavenger, in the culture medium prompted reduction in number of vacuoles in F-HABP07 along with drop in ROS level, implying that excess ROS generation triggers initiation of autophagic vacuole formation prior to apoptosis due to overexpression of HABP1. Thus, the phenomenon of autophagy takes place prior to apoptosis induction in the HABP1 overexpressing cell line, F-HABP07.

## Introduction

The glycosaminoglycan, hyaluronan (HA), an endogenous antioxidant [1-3] regulates several cellular pathways via a battery of HA binding proteins or ‘hyaladherins’ [4]. Depolymerization of HA takes place mainly by OH**^˙^** attack at multiple sites within the HA structure and the products formed finally cause the cleavage of glycosidic bonds through β-scission generating HA oligomers [5,6]. HA oligomers of 2.5 X 10^3^ have been observed to inhibit anchorage independent growth of tumor cells and to induce apoptosis [7] although polymeric HA has been implicated in cellular proliferation [8,9]. Hyaluronan-binding protein 1 (HABP1), a 34 kDa glycosylated phosphoprotein and member of the ‘hyaladherin’ family of proteins, interacts with hyaluronan (HA) [10] and is implicated in cell signaling. HABP1 shows a complete cDNA sequence identity with p32, a protein co-purified with the splicing factor SF2 and gC1qR, the human receptor for the globular head of the complement factor 1q [11-13], thus represented as HABP1/p32/gC1qR in the Human Genome. The gene encoding HABP1 is located in chromosome 17p12-p13 having GenBank ID: AF275902 [14]. The crystal structure of HABP1/p32 reveals it as a trimer, with the three monomers forming a doughnut shaped quaternary structure with an asymmetrical charge distribution on the surface surrounding a central channel [15]. Although HABP1 primarily localizes in the mitochondrial matrix but its presence has also been reported at the cell surface, nucleus and cytosol suggesting myriad ligand binding capacity attributing to its multifunctional nature [9,16-18]. The sequence analysis of HABP1 revealed several sites for CKII phosphorylation and a substrate site for MAPK [^160^PELTSTP^166^]. HABP1 has been reported as an endogenous substrate for MAPK and upon mitogenic stimulation, translocates to the nucleus in a MAPK dependent manner in HeLa and F111 cells [19]. Constitutive expression of HABP1/p32/gC1qR in normal fibroblasts perturbs its growth characteristics and induces apoptosis at 60 h as indicated by increased Bax expression and TUNEL positive cells [20]. Further, it was observed that HABP1 accumulates in the mitochondria leading to generation of Reactive Oxygen Species (ROS), increased Ca^++^ influx in mitochondria resulting in membrane potential drop creating mitochondrial dysfunction [16]. On the contrary, HepG2 cells, containing an endogenously high level of an array of antioxidant enzymes stably overexpressing HABP1 do not lead to ROS generation, cellular stress and apoptosis rather are more proliferative in nature with increased polymeric HA than their parent cell line [9].

ROS, considered as a metabolic stress and autophagy inducer, acts as a buffering mechanism under such stresses by way of metabolizing nutrients resulting from macromolecular degradation. Two major degradative routes in eukaryotes which maintain homeostasis are the proteasomal and lysosomal systems. The lysosomal or autophagosomal system is responsible for the degradation of long-lived proteins, which represents the vast majority of cellular proteins and for the turnover of organelles [21-24]. The highly reactive nature of ROS attributes to both its destructive properties and also its functioning as a signaling molecule. Increased ROS level has been suggested to be one of the key regulators of autophagy by several studies [25-28] and attempts are being made to unfold the role of autophagy in oxidative stress-induced cell death. Class III PI 3-kinase has been implicated in ROS production resulting in stimulation of autophagy [25]; while, several studies in the past have suggested class I PI3-kinase as an inhibitor of autophagy [29,30]. One of the regulators of autophagy is the tumor suppressor Beclin 1, homologue of yeast Apg6 which forms a complex with class III PI3 kinase and reportedly induces autophagy [31,32]. smARF, the product of internal initiation of translation of the tumor suppressor p19ARF/p14ARF acts as autophagy inducer by generation of ROS upon translocation to mitochondria. The mitochondrial translocation of this short-lived protein occurs only upon physical interaction and stabilization by HABP1/p32; otherwise, it is short-lived due to proteasomal degradation [33,34]. 

Excess ROS in cell can trigger both autophagy and apoptosis [27,28,16] and F-HABP07 cells have been already reported to generate numerous vacuolar structures and to undergo apoptosis at 72 h of growth [16,20]. The nature of the vacuolated structures is unknown and moreover HABP1 has been perceived to have a role in the induction of autophagy by interaction with smARF. Thus, our interest is to explore the regulatory role of HABP1 overexpression on autophagic vacuole formation dependent on ROS generation and whether it can be modulated with PI3 kinase inhibitor and endogenous antioxidant. 

## Materials and Methods

### Materials

Chemicals including the reagents required for cell culture were obtained from Sigma Aldrich Chemicals Pvt. Ltd. (USA). Primary as well as secondary antibodies were obtained from Santa Cruz Biotechnology Inc. (USA), Sigma Aldrich Chemicals Pvt. Ltd. (USA), Abcam (USA) and Cell Signaling Tech. (USA). Alexa Fluor 488 and 546, Streptavidin Alexa Fluor 568 were from Molecular Probes Inc. (Eugene, OR). Oligomeric HA, 12mer (o-HA) was obtained from Seikagaku Corporation. Low molecular weight marker was obtained from Amersham, UK. Cell culture plastic wares were obtained from Corning-Costar Inc. (Corning, NY, USA). 0.22µM membrane filters for filtering media and PVDF membranes were obtained from Millipore (MA, USA). Filtration unit and isopropanol cryobox were purchased from Nalgene (NalgeNunc International Corporation, Rochester, NY, USA). Water used for preparing media and reagents was either autoclaved triple distilled (distilled in our laboratory) or autoclaved Milli Q (obtained from water purification system, Millipore, MA, USA). 

### Maintenance of Cell Line

Cell lines F111, F-pCDNA01 and F-HABP07 [20] were cultured in Low Glucose DMEM (Dulbecco’s Modified Eagle’s Medium), containing 1gm/L glucose. Media was supplemented with 10% FBS, 100μg/ml streptomycin and 50μg/ml fungizone in tissue culture flasks and dishes. Cultures were grown at 37°C in a humid atmosphere with 5% CO_2_ and 95% air. Cells were routinely maintained in monolayer culture and detached from the plastic surfaces of tissue culture wares by trypsinization (trypsin-EDTA treatment: 0.25% trypsin and 2mM EDTA in 0.01M PBS, pH 7.2) for regular sub-culturing. 

### Transmission Electron microscopy

 The cell lines grown for 60 h were washed thrice with PBS and fixed in 3% glutaraldehyde at 4°C for minimum of 4 h to overnight. After washing, cells were again fixed for 2 h in 1% osmium tetraoxide in phosphate buffer at 4°C. After several washes with PBS, the cells were dehydrated in graded acetone solutions and embedded in CY212 araldite resin. Ultra-thin sections of 60-80 nm thickness were generated using Ultracut E Ultramicrotome and the sections were stained with alcoholic uranyl acetate and lead citrate for appropriate time intervals. The grids were then examined with Transmission Electron Microscope (Morgagni 268 Model, Philips) operated at 80kV. 

### Detection of Autophagic vacuoles by staining of cells with Monodansyl Cadaverin (MDC)

The development of autophagic vacuoles was studied at different time points (36, 48, 60 and 72 h) by staining cells with 0.05mM MDC. MDC specifically binds to Phosphoethanol Amine (PE) in the autophagic membrane [35]. Cells after completion of the specified time points were washed with PBS (pH 7.2) to remove culture medium. Cells were then incubated with 0.05mM MDC in PBS at 37°C for 10 min. After incubation cells were washed 4 times with PBS and immediately analyzed by fluorescence microscopy [Excitation wavelength=335nm; Emission wavelength = 525nm]. 

### Cell Treatments

For 3-Methyl Adenine (3-MA) treatment cells were grown on sterile coverslips in a 12-well plate for different time points. After completion of the time point, the media was aspirated out from the cells, replenished with fresh culture media containing different concentrations of 3-MA (0, 0.1, 1 or 10mM) and then incubated for 30 min at 37°C. Cells were then washed 4 times with PBS (pH 7.2), fixed with chilled methanol and kept at -80°C. 

F111 and F-HABP07 cells were treated with the potent vacuolar H^+^ ATPase inhibitor bafilomycin A1 (Baf A1) to assess whether the increased autophagosomes during 36 and 48 h in F-HABP07 cells were due to autophagic degradation or inhibition of autolysosome formation as per the methodology suggested by Rubinsztein et al [36]. The cells were treated for 6 hrs with 50nM Baf A1 at 36 and 48 h of growth and then harvested for immunoblotting to compare the accumulation of MAP-LC3-II in the treated and untreated cells. F-HABP07 cells had earlier shown increased MAP-LC3-II accumulation during the abovementioned time periods. 

In order to check whether supplementation of HA has any effect on the cellular growth and morphology, cells were grown in 6 well plate for ROS Assay and MDC staining, 24 well culture dishes for MTT assay and 12 well culture dishes for haematoxylin – eosin (H-E) staining. Cells were supplemented with 150µg/ml of polymeric HA and o-HA at 36 h, grown till 60 h and then either subjected to ROS Assay, cell survivability (MTT) Assay or H-E staining.

### Assay of intracellular ROS

Intracellular H_2_O_2_ production was detected by fluorescence of 2',7'-dichlorodihydrofluorescein diacetate, acetyl ester, H_2_DCFDA (10 µM) incubated under various conditions for 10 min in dark as previously reported [16].

### Cell survivability Assay

Equal number of cells was seeded in each well of a 24 well culture cluster. The samples were collected at required time point in triplicates. 10 µl of the yellow tetrazole dye [3-(4,5-Dimethylthiazol-2-yl)-2,5-diphenyltetrazolium bromide], MTT (1mg/ml) was added to each well and incubated at 37°C for 4 h in a humidified CO_2_ incubator. The precipitate formed was solubilized in 150 µl of the solubilization buffer, dimethyl sulfoxide (DMSO) after discarding the media and then the absorbance was recorded at 570 nm.

### Morphological Analysis by Haematoxylin – Eosin (H-E) Staining

 Cells grown for requisite time were fixed in chilled methanol and processed for H-E Staining. Methanol was aspirated out followed by two changes of 99% ethanol for 2 min each. Then two changes of 95% ethanol for 2 min each were given. After washing with double distilled water for 1 min haematoxylin was put on the coverslips and kept for 1 min. After thorough washing in running water for 3 min, Eosin was put and kept for 30 sec only. Cells were then rinsed in running tap water for 30 sec. Dehydration steps were followed by giving two changes of 95% ethanol for 2 min each and again two changes of 99% ethanol for 2 min each. At last coverslips were mounted with 50% glycerol sealed with nail enamel and observed under phase contrast microscope (Nikon) fitted with Nikon FX-35W camera.

### Immunofluorescence study of cells

Indirect immunofluorescence staining of the cells was done by fixing the cells at specific time periods with paraformaldehyde (2%) and the cells were made permeable with glycine (0.1M) and Triton X-100 (0.1%). All the chemicals and antibody dilutions were prepared in PBS (pH 7.2). After blocking with BSA (3%), the cells were incubated with specific primary antibody for 2 h. Secondary antibody raised against either mouse or rabbit were used for detection of proteins. These were fluorescence tagged either with Alexa Fluor 488 or 546 (Molecular Probes Inc., USA). To analyze nuclear morphology, DAPI (5mg/ml) (Sigma, USA) was added to the cover slip 15 min before secondary antibody washing. Thereafter, the cells were washed and mounted in 50% Glycerol in PBS. Fluorescence images were monitored using an Axioscope microscope (Carl Zeiss, Germany) equipped with epifluorescence and Axiocam camera system coupled with Axio Vision software (Carl Zeiss, Germany).

Immunostaining for HA was done by processing the cells as mentioned above for immunofluorescence study till the blocking stage. Cells were then incubated with commercially available Biotinylated HABP for 1 h, washed with PBS and incubated with secondary antibody Streptavidin conjugated with Alexa Fluor 568 (1:300) for 1 h. Rest of the steps are similar to as mentioned above.

### Immunoblot analysis

The protein samples electrophoresed by SDS-PAGE were electro-blotted on either nitrocellulose membrane or PVDF by applying 0.8mA/h current in a semi-dry transfer unit or wet-transfer unit. Following transfer the membrane was blocked with 5% non-fat dry milk in TBS for 2 h at room temperature and incubated with desired primary antibody for overnight at 4°C. PVDF membrane was then washed three times with TBST (TBS with 0.1% Tween-20) and incubated overnight with primary antibody, washed, then incubated for 1 h with horseradish peroxidase or alkaline phosphatase conjugated secondary antibody. The bound antibody complexes were detected using the NBT/BCIP system or enhanced chemiluminescence (ECL) system. Primary antibody dilutions used are 1:1000 for Beclin 1, MAP-LC3, DRAM, PTEN and p53, while dilution of 1:20,000 and 1:5000 were used for GAPDH and Tubulin respectively.

### Detection of depleted and depolymerized HA and separation on a gradient polyacrylamide gel

 Polyacrylamide gel electrophoresis method for molecular mass determination of HA [37] was used after separation of HA fraction from cultured cells using a procedure described earlier [9]. Equal number of F111 and F-HABP07 cells were seeded in culture dishes and allowed to grow for 60 h after which the media was removed and the monolayer washed gently with PBS. Subsequently, the cells were treated with 200 µl of 50 mM sodium acetate (pH 6.0) containing 250 µg/ml proteinase K, 5 mM EDTA and 5 mM cysteine. After incubating for 10 min at 37°C, the cells were scraped and collected into micro-centrifuge tubes followed by incubation for 5 h at 60°C. Inactivation of Proteinase K was carried out by incubation in a boiling water bath for 10 min followed by centrifugation at 13,000 rpm. Supernatant was collected and treated with 4 volumes of 1% cetylpyridinium chloride in 20 mM NaCl, for 1 h at room temperature and centrifuged at 13,000 rpm for 15 min. After discarding the supernatant, precipitate was washed with 1 ml water, centrifuged again and dissolved in 50 µl of 4M guanidine-HCl. 900 µl of ethanol was added to the solution and the tube was kept at -20°C for an hour after which each sample was centrifuged and precipitate retained and dissolved in 50 µl of 50 mM sodium acetate (pH 6.7). This fraction was used as undigested sample. Aliquots of equal volume from these were then processed for hyaluronan digestion using 50 µg/ml of bovine testicular hyaluronidase (BTH) for 1 h at 37°C. Equal volumes of both undigested and BTH- digested products derived from two cell lines were loaded onto a 5-20% gradient gel as described previously [37], along with BTH-digested and undigested commercially available polymeric HA from human umbilical cord acting as positive controls. The gel was then stained with 1% Alcian blue in 3% acetic acid, destained and subsequently stained with silver nitrate [37].

### Statistical analyses

ImageJ software was used to calculate the relative fold change in the immunoblots. The average values of three observations are denoted in the figures. Assay results were analyzed by either One-Way ANOVA or Student’s T-test when applicable to evaluate differences between control and the test sample. Significance level was set at p<0.05, unless otherwise mentioned.

## Results

### Overexpression of HABP1 in normal Fibroblasts induces formation of autophagic vacuoles

**Figure 1:**
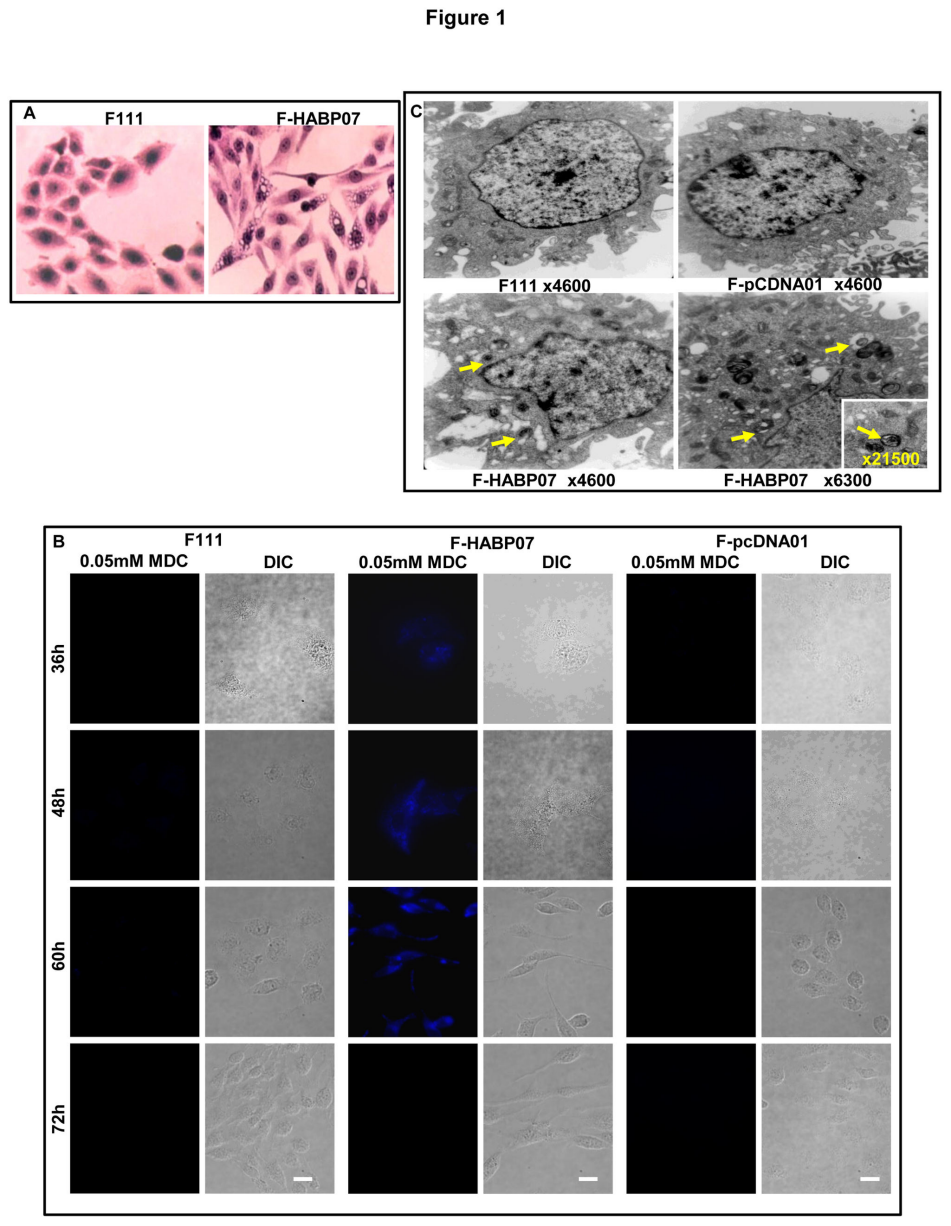
Characterization of vacuolation in HABP1 overexpressing fibroblast (F-HABP07). [A] ***Alteredmorphologicalappearance***-- Visualization of numerous vacuoles in F-HABP07 cells at 48 h upon Haematoxylin-Eosin staining, under light microscope (40X Magnification). [B] ***IdentificationofautophagicvacuolesusingMDCstaining***-- F111, F-HABP07 and F-pCDNA01 cells grown for different time points were stained with the auto-fluorescent compound, MDC (0.05mM) which specifically stains autophagic vacuoles, confirms autophagic vacuolation at 36, 48 and 60 h of growth in F-HABP07 cells only. Scale=10µ. [C] ***TransmissionElectronMicroscopy***-- Ultrastructure confirms autophagic vacuole (yellow arrow) generation in F-HABP07 cells with respect to parental control F111 and vector control F-pCDNA01. Magnification (x) of ultramicrograph has been mentioned in each corresponding figure.

The stably transfected fibroblast (F-HABP07) have already been reported [16,19] to have altered morphology with numerous vacuolar structures. The nature of these vacuolar structures was not identified previously. Figure **1A** shows the morphological difference between the normal and transformed fibroblast upon H-E staining. The auto-fluorescent compound, MDC was used for intracellular labeling of autophagic vacuoles. MDC specifically binds to phosphatidylethanol amine (PE) in the autophagic vacuolar membrane. MDC labeled organelles contain the lysosomal enzyme acid phosphatase and the mature form of cathepsin D. The development of autophagic vacuoles at different time points (36, 48, 60 and 72 h) was studied by staining live cells with 0.05mM MDC. Upon fluorescence microscopic analysis it was found that HABP1 overexpressing F-HABP07 cells at 36, 48 and 60 h of growth show positive MDC staining (Figure **1B**) and not at 72 h of growth, rather undergo into apoptosis as previously reported [16]. Both the normal fibroblasts (F111) and vector transformed fibroblast (F-pCDNA01) do not show any positive MDC staining. Transmission electron micrographs of the cell lines F111, F-HABP07 and F-pCDNA01 cultured for 60 h revealed the presence of autophagic vacuoles only in F-HABP07 cells as indicated with yellow arrows in Figure **1C**.

### Increased expression of autophagic markers Beclin 1 and MAP-LC3-II in F-HABP07 cells

**Figure 2:**
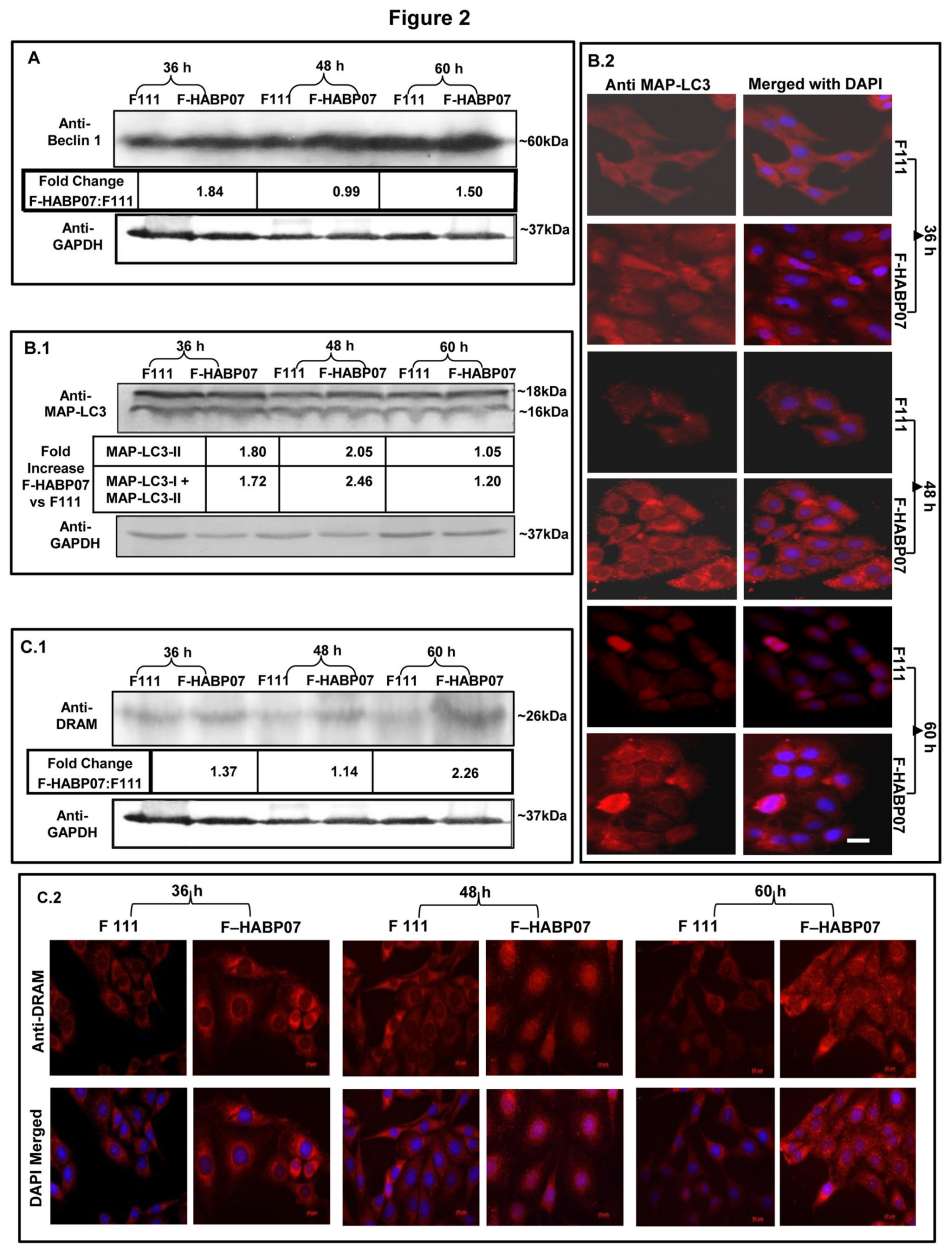
Detection of autophagic markers by immunoblotting and immunocytochemistry. [A] ***ElevatedexpressionoftheautophagicmarkerBeclin 1uponoverexpressionofHABP1infibroblasts***-- Whole cell lysates of F111 and F-HABP07 grown for 36, 48 and 60 h were subjected to immunoblot analysis, using antibody against the autophagy modulator, polyclonal Beclin 1 (1:1000) and monoclonal anti-GAPDH (1:20000) as control. Upon normalization of expression of Beclin 1 against GAPDH, Beclin 1 was found to be much higher in F-HABP07 at 36 and 60 h of growth, but it reduces at 48 h of growth to almost basal level. [B] ***ElevatedexpressionoftheautophagicmarkerMAP-LC3uponoverexpressionofHABP1infibroblasts***-- Immunoblot analysis confirming a higher expression of the autophagic marker MAP-LC3-II at 48 h of growth in F-HABP07 as compared to F111 (**B**.1) after normalization of MAP-LC3-II expression with GAPDH expression. Upsurge in MAP-LC3 expression in HABP1 overexpressing fibroblasts, F-HABP07 compared to F111 (grown for 36, 48 and 60 h) was observed upon visualization under Fluorescence microscope after immunocytochemical analysis performed using MAP-LC3 antibody (1:200), reprobing with anti-rabbit Alexa Fluor 546 and DAPI for staining the nucleus (**B**.2). Scale=10µ. [C] ***IncreasedexpressionofautophagicmodulatorDRAM***-- Immunoblotting with antibody against the lysosomal protein DRAM, which is transactivated by p53 and normalization with GAPDH by the help of ImageJ. The fold increase calculated for each time point (F-HABP07 versus F111) indicates that the expression level of DRAM was higher at 36 and highest at 60 h of growth (**C**.1). Similar expression and nuclear translocation in F-HABP07 cells was also observed after immunocytochemical analysis (**C**.2).

After confirming the presence of autophagic vacuolation by MDC staining, the expression profile of the autophagic marker Beclin 1 was verified. Beclin 1, a homologue for Apg6 and an important member of the initiation complex for autophagic machinery is reported to be mono-allelically deleted in several cancers. Whole cell lysates of F111 and F-HABP07 cells grown for 36, 48 and 60 h were immunoblotted with Beclin 1 antibody and GAPDH antibody as an internal control. After normalization with the respective GAPDH expression, the F-HABP07 cells were found to have a much higher expression of Beclin 1 at 36 h and 60 h of growth than its normal counterpart. On the contrary, its expression decreased to almost basal level at 48 h of growth, when more number of vacuoles was observed in the transformed cells (Figure **2A**). In continuation, we checked the level of expression of another autophagy marker, the lipidated form of microtubule associated protein light chain 3 (MAP-LC3-II). MAP-LC3, a homologue of Apg8 is essential for autophagy and is found to be associated with autophagosome membrane after processing. When whole cell lysates from F111 and F-HABP07 grown for 36, 48 and 60 h were subjected to immunoblot analysis (Figure 2B.1) using MAP-LC3 antibody, it showed a much higher expression of MAP-LC3-II at 48 h of growth. This coincided with the observation of maximally generated vacuoles at that time point. Immunocytochemical analysis (Figure 2B.2) also showed a higher expression of the protein MAP-LC3 in F-HABP07 cells at all the time points. The expression of the lysosomal protein DRAM, known as an autophagy modulator was also observed to be higher in F-HABP07 cells compared to F111 throughout the three time periods. But expression of DRAM is higher during 36 h and highest at 60 h of growth, coinciding with the induction of apoptosis (Figure 2C.1). The immunocyochemical analysis (**Figure 2C.2**) also indicates a similar expression as observed in the western blot experiment. 

### Reduction of vacuole formation is transiently effective with PI3 kinase inhibition

**Figure 3:**
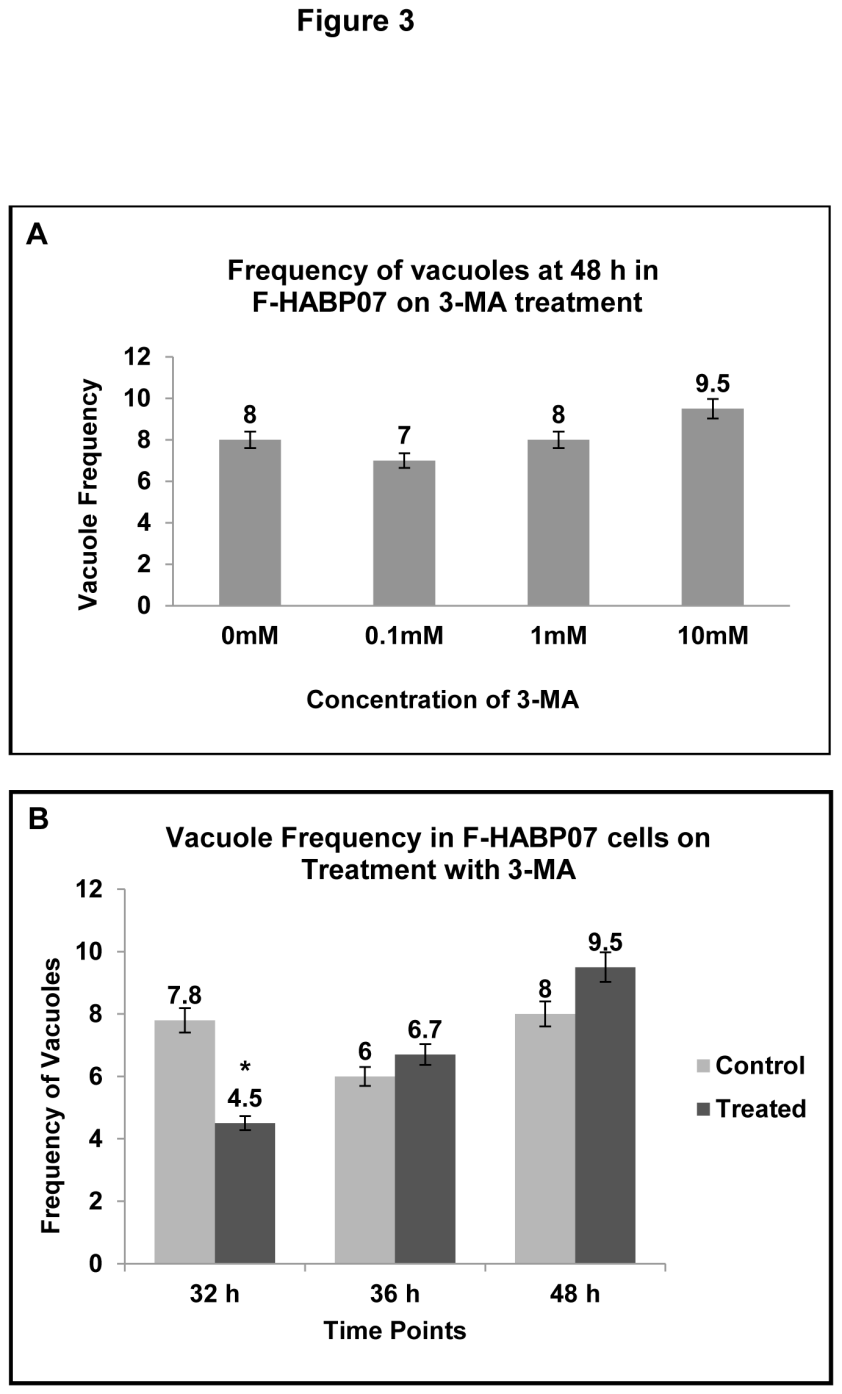
Vacuole Frequency is affected at 32 h upon 3-MA treatment in a time dependent manner. [A] ***ConcentrationindependentoccurrenceofvacuolesinF-HABP07at 48h*** -- Vacuole Frequency counted in F-HABP07 at 48 h after treatment with varying concentration of 3-MA (0mM, 0.1mM, 1mM and 10mM) and H-E staining depicted in the histogram after counting the number of vacuolated cells out of the total cells in 10 sites and then calculating the vacuole frequency per 100 cells. Single factor one-way statistical analysis of variance carried out using ANOVA software, with difference at a level of *p ≤ 0.05 between groups considered as statistically significant, indicated insignificant change in the vacuole frequency upon the aforementioned treatment. [B] ***Reductionofvacuolefrequencyupontreatmentat 32h--***Vacuole frequency in F-HABP07 counted after treatment with PI3 kinase inhibitor 3-MA and then H-E staining, as mentioned before, shows that it is effective in reduction of vacuole formation at 32 h by almost 40% and not beyond that time point. The change in vacuole frequency at 32 h upon treatment with 3-MA was found to be significant and has been indicated in the figure [*].

It has already been reported that both ROS and PI3 kinase play an important role in autophagic vacuole formation. Class III PI3 kinase which forms a part of the initiation complex is in fact involved with ROS generation, while class I PI3- kinase has been reported to inhibit autophagy. In order to check how the PI3-kinase pathway is involved in the autophagic vacuolation in the F-HABP07 system, cells were subjected to 3-Methyl Adenine (3-MA) treatment. 3-MA, a known PI3 kinase inhibitor, is also a classical inhibitor of the autophagic pathway. As the F-HABP07 cells were observed to have the highest amount of vacuoles with about 6 fold increase in ROS at 48 h of growth, they were subjected to treatment with 0.1mM, 1mM and 10mM of 3-MA for 30 min. After the treatment cells were stained with haematoxylin and eosin and then subjected to light microscopy and vacuoles were counted. Figure **3A** demonstrates that vacuole frequency per 100 cells was not significantly affected for F-HABP07 cells treated with different concentrations of 3-MA as compared to the control cells. In order to examine whether 3-MA can affect autophagic vacuolation at earlier time point, when the level of ROS was lower, F-HABP07 cells were subjected to treatment with 10mM 3-MA for 30 min at 32, 42 and 48 h. After performing H-E staining vacuoles were counted in F-HABP07 cells. Vacuole frequency in F-HABP07 per 100 cells counted after treatment with PI3-kinase inhibitor 3-MA and then H-E staining revealed that the vacuole frequency significantly decreased by about 40% on treatment at 32 h, but remained unaffected at the other time points (Figure **3B**). Thus, 3-MA is effective in reduction of vacuole formation up to 32 h and not beyond.

### Treatment with bafilomycin A1 reveals augmented autophagic degradation taking place in F-HABP07 cells

**Figure 4:**
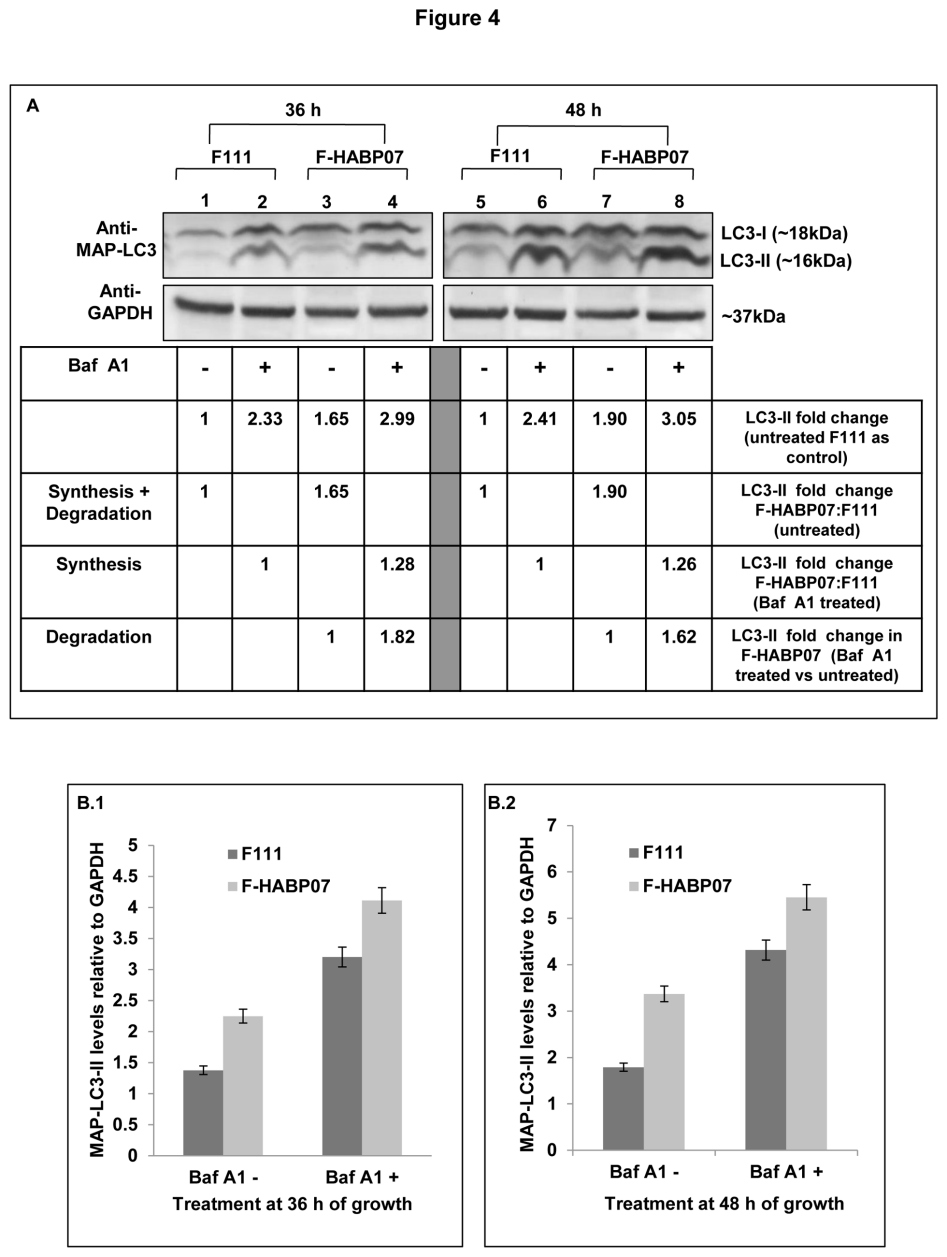
Increased autophagic flux or lysosomal proteolysis upon HABP1 overexpression verified by Baf A1 treatment. [A] ***ImmunoblottingofBafA1treatedcellsrevealbothsynthesis and degradationinF-HABP07***-- Lysosomal proteolysis was inhibited by Baf A1 (50nM) treatment at 36 h (Lane 1-4) and 48 h ((Lane 5-8)) and changes in MAP-LC3-II levels were observed in both treated (6 h) and untreated F111 and F-HABP07 cells in order to access autophagic degradation. After normalization with GAPDH fold change of MAP-LC3-II (16kDa) was calculated taking its expression in untreated F111 as control, using ImageJ. Amount of synthesis plus degradation upon HABP1 overexpression was measured from the untreated controls i.e. Lane 3 vs Lane 1 (36 h) and Lane 7 vs Lane 5 (48 h). This revealed increased expression in F-HABP07 compared to F111 cells, during 36 and 48 h of growth as per the earlier observation. Comparison between the Baf A1 treated samples i.e Lane 4 vs Lane 2 (36 h) and Lane 8 vs Lane 6 (48 h) gave an idea about autophagosome synthesis. It indicated an augmented synthesis of autophagosome in F-HABP07 cells. Increased autophagic flux or degradation in F-HABP07 is divulged from fold increase in MAP-LC3-II expression in Baf A1 treated and untreated cells i.e Lane 4 vs lane 3 (36 h) and Lane 8 vs Lane 7 (48 h). The fold changes have been indicated in a tabulated form. [B]***Graphicalrepresentationoffoldchanges***-- The fold changes for MAP-LC3-II expression after normalization with GAPDH in Baf A1 treated (Baf A1 +) and untreated (Baf A1 -) F111 and F-HABP07 cells have been represented graphically for both 36 h (**B**.1) and 48 h (**B**.2).

MDC staining positive for autophagosomes have been observed in F-HABP07 cells during 36, 48 and 60 h of growth with increased expression of MAP-LC3-II during 36 and 48 h compared to F111 cells. Increased autophagosomes can result from either increased autophagic flux or delayed trafficking to the lysosome, reduced fusion between both compartments and impaired lysosomal proteolytic activity [36]. Hence, in order to elucidate whether autophagic degradation is actually taking place in the HABP1 overexpressed fibroblasts, both the F111 and F-HABP07 cells were subjected to Baf A1 treatment, according to the protocol suggested by Rubinsztein and group [36]. F-HABP07 cells upon Baf A1 (50nM) treatment for 6 h given at 36 and 48 h of growth revealed an increased expression of MAP-LC3-II as evident from the fold increase (Figure **4A**). Elevated level of MAP-LC3-II in F-HABP07 cells, similar to what has already been observed is also evident from the immunoblot analysis of the samples from Baf A1 untreated F111 and F-HABP07 cells after normalization with GAPDH (Figure **4A** and Figure **4B**). The above observation revealed an upsurge in autophagic flux in normal fibroblasts upon HABP1 overexpression.

### Treatment with proteasome inhibitor MG132 leads to cell death in HABP1 overexpressing fibroblasts

**Figure 5:**
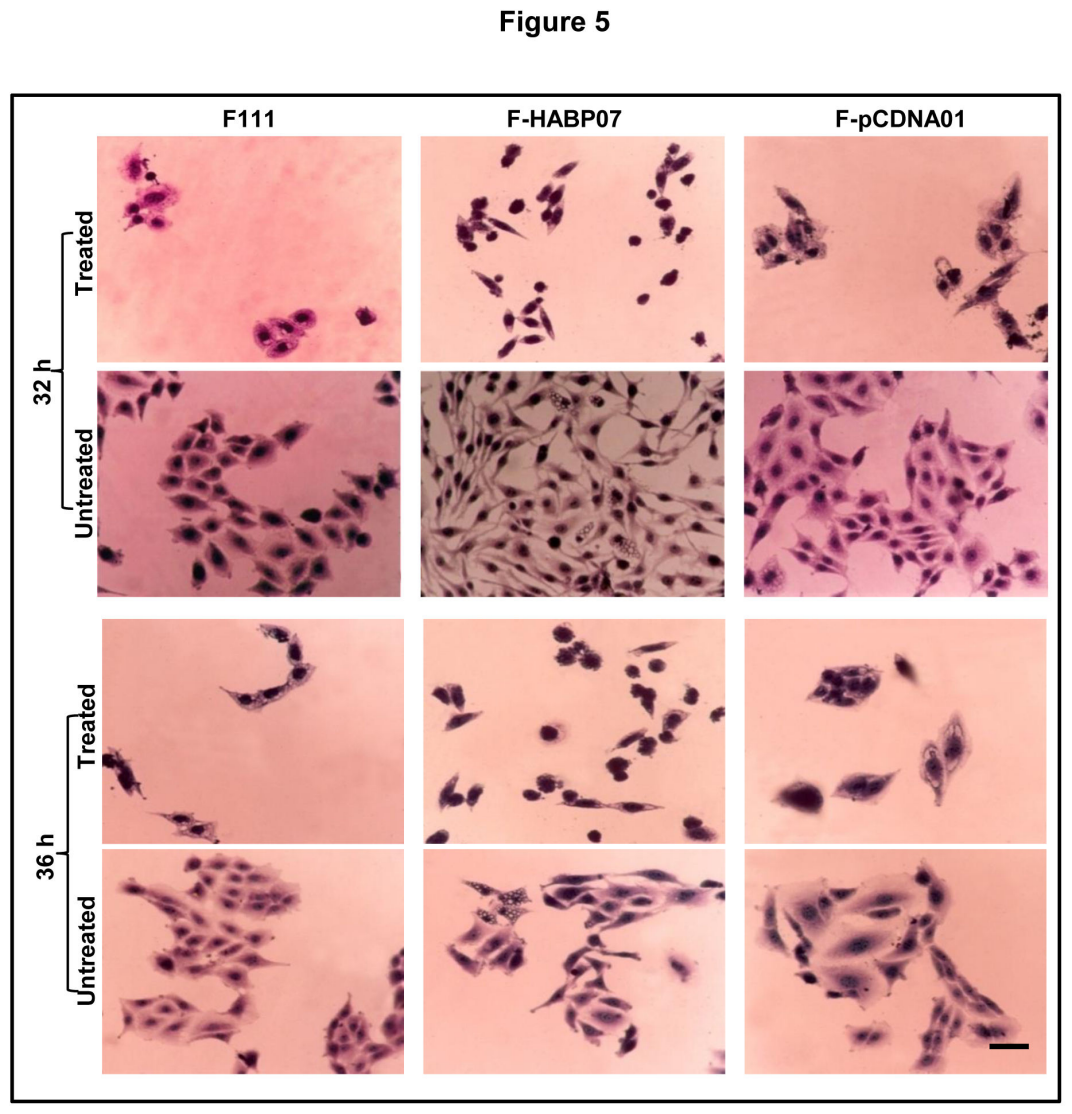
Proteasome inhibitor MG132 leads to cell death in HABP1 overexpressing fibroblasts. Haematoxylin - Eosin staining after treatment of F111, F-HABP07 and F-pCDNA01 with proteasome inhibitor MG132 (5mM, 12 h) both at 32 h and 36 h leads to cell death in HABP1 overexpressing fibroblasts while the normal and the vector transformed fibroblasts showed generation of vacuoles. Scale=10µm .

Cells have two degradative pathways for elimination of misfolded proteins and turnover of raw materials, the proteasomal degradation pathway and the autophagic machinery. When proteasomal pathway is blocked the cells tend to compensate by the other pathway. In order to examine the effect in the cells generating autophagic vacuoles, the three cell lines F111, F-HABP07 and F-pCDNA01 were treated with the known proteasome inhibitor MG132 (5mM, 12 h) at 32 h and 36 h of growth and then processed for H-E staining. On microscopic analysis it was observed that the F-HABP07 cells undergo cell death in both the cases, while the F111 and F-pCDNA01 cells exhibit ramifications in the form of many vacuolar structures (Figure **5**), suggesting that on inhibition of proteasomal degradation in F111 and F-pCDNA01, the cells might be undergoing autophagic degradation. But, F-HABP07 cells which generated autophagic vacuoles due to excess ROS could not undergo additional autophagic survival stimuli, rather went into cell death.

### Upregulation of tumor suppressors and tumor suppressor related proteins in the HABP1 transformed fibroblast F-HABP07

**Figure 6:**
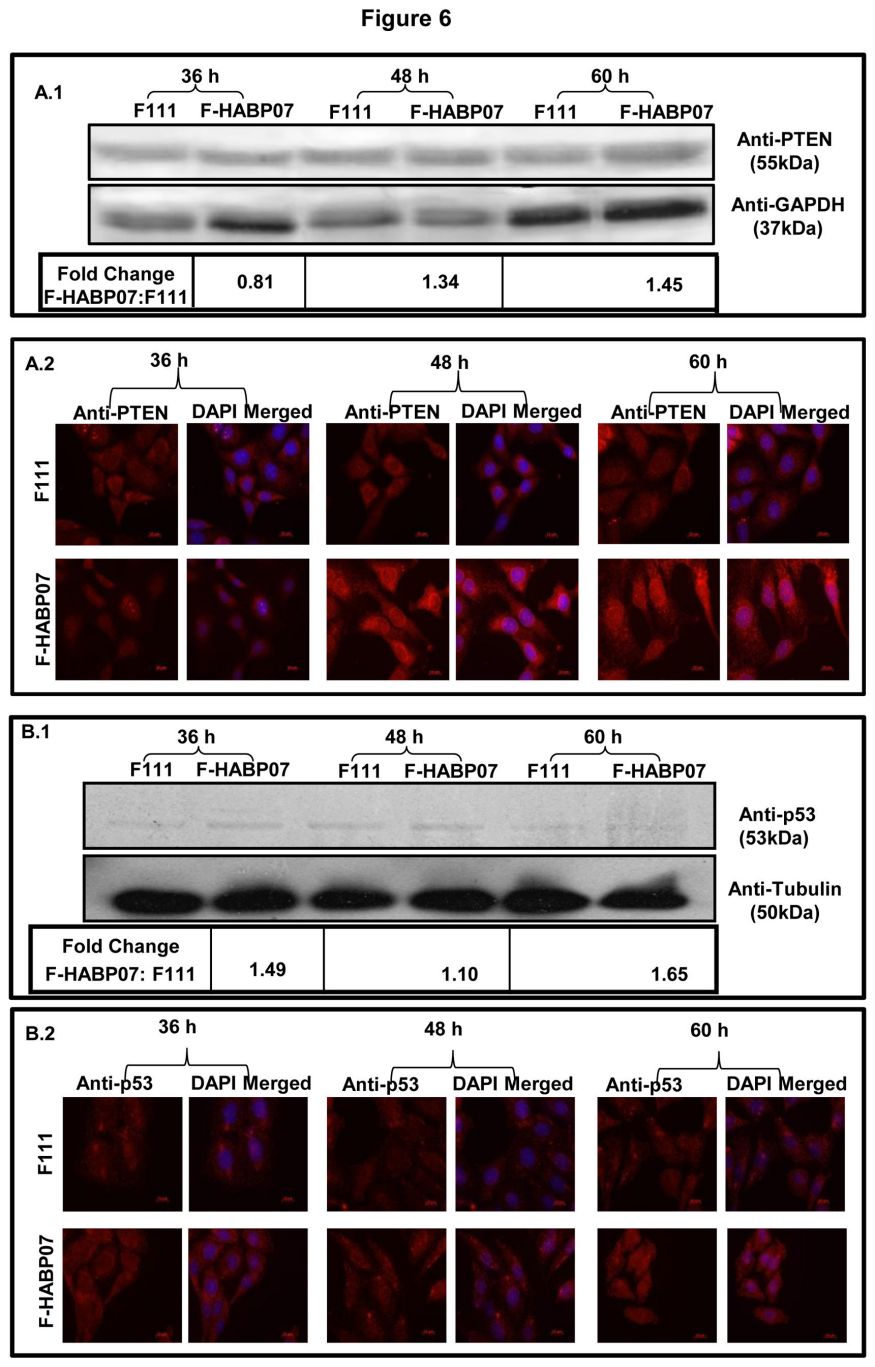
Upregulation of tumor suppressors in the HABP1 transformed fibroblast F-HABP07. Whole cell lysates of F111 and the HABP1 transformed fibroblast cells (F-HABP07) were resolved by SDS-PAGE, transferred and immunoblotted with antibodies for the tumor suppressors PTEN (**A**.1) and p53 (**B**.1). Expression of PTEN which was initially lower in F-HABP07 gradually increased with progression of growth from 36 to 60 h. This coincides with the immunocytochemical data which also show nuclear localization of PTEN at 48 and 60 h of growth (**A**.2). p53 expression increased in F-HABP07 cells at 60 h of growth. Immunocytochemical analysis of F111 and F-HABP07 cells grown for the three time periods shows a higher expression and nuclear localization of p53 at 60 h of growth, indicating an activated state in F-HABP07 cells (**B**.2).

Whole cell lysates prepared from F111 and the HABP1 transformed fibroblast cells (F-HABP07) were resolved by SDS-PAGE, transferred and immunoblotted with antibodies for the tumor suppressors PTEN and p53. The fold increase has been calculated for each time point (F-HABP07 versus F111) and represented after normalization with GAPDH and tubulin respectively by the help of ImageJ. Immunocytochemical analysis was also done for the two cell lines grown for the above mentioned time periods with antibodies against PTEN and p53. Results indicate that the expression of PTEN increased in F-HABP07 cells with increase in time of growth from 36 to 60 h, along with nuclear translocation of the tumor suppressor at 48 and 60 h of growth (Figure 6A.1 and Figure 6A.2). Expression of the other tumor suppressor p53 was found to be higher at 36 and 60 h in F-HABP07 cells compared to F111 cells (Figure 6B.1). Immunocytochemical analysis also showed a higher expression and nuclear localization of p53 in F-HABP07 at 60 h of growth (Figure 6B.2).

### Hyaluronan depolymerization and its depletion in excess ROS generating HABP1 overexpressing fibroblast

**Figure 7:**
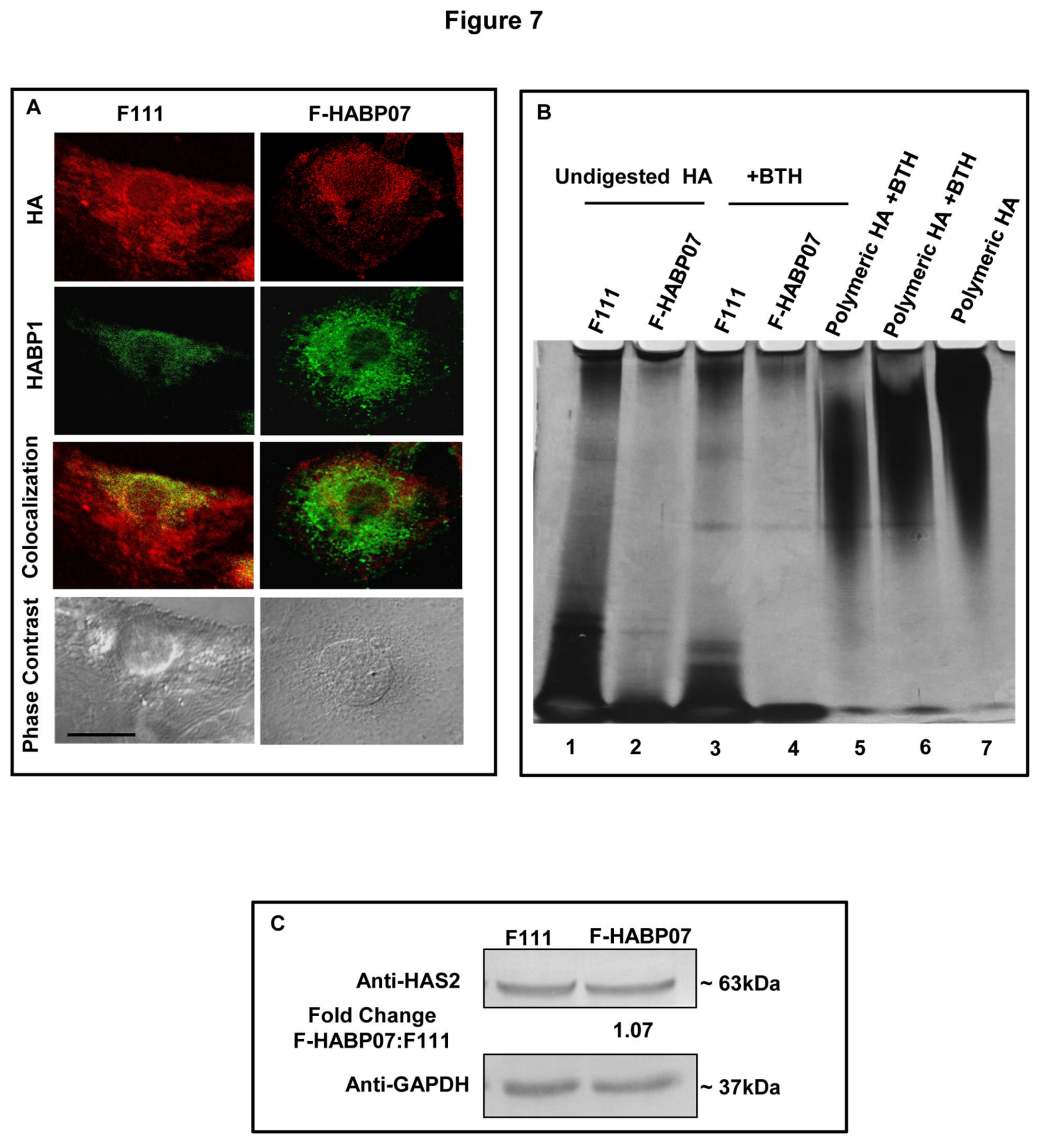
Depolymerized and depleted HA in excess ROS generating HABP1 overexpressing fibroblasts. [A] ***Colocalization studyofHAwithHABP1*** -- Cytotochemical staining for hyaluronan was performed in normal F111 and F-HABP07 using biotinylated hyaluronan binding protein and probed with streptavidin conjugated with Alexa Fluor 568. The depletion of HA polymer resulting from oxidant release by HABP1 overexpression in F-HABP07 cells (column 2) with respect to parental control cells (column 1) can be explained since HA polymer is known to be most susceptible to ROS-mediated degradation to HA oligosaccharide. Row 4 represents the morphology of two cell lines by phase contrast microscopy. Scale bar represents 10μm for all. [B]***Detectionofdepleted and depolymerizedHAinF-HABP07--*** HA was purified from both F111 and F-HABP07 cells and then subjected to digestion with 50µg/ml of BTH. Equal volumes of both the undigested (lane 1 and 2) and BTH-digested products from the two cell lines (lane 3 and 4) were electrophoresed onto a 5–20% gradient polyacrylamide gel. Commercially available polymeric HA (lane 7) and two different concentrations of the same polymeric HA treated with BTH (lane 5 and 6) were taken as controls. The gel was stained with 1% Alcian blue in 3% acetic acid and then subsequently with silver nitrate. A significantly reduced level of polymeric HA in F-HABP07 cells is clearly evident as compared with the F111 cells and the high molecular weight HA further disappears upon subsequent treatment with BTH. [C]***DetectionofHAS2level***-- Western blotting performed on cell lysates of F111 and F-HABP07 grown for 60 h revealed that the expression level of HAS2 is similar in both the cell lines.

Several reports suggest that HA, which is abundantly available in biological fluids gets degraded in pathologic conditions, probably because of inflammation induced free-radical mediated depolymerization of the HA chain [38-40]. Depolymerization of HA induced by free radicals is a well-characterized phenomenon where HA acts as a scavenger of ROS [41]. Thus, our observation of excess ROS generation due to mitochondrial HABP1 accumulation [16] prompted us to compare the profile of HA and HABP1 in the three cell lines. Cytochemical staining of HA and HABP1 in the Figure **7A** in normal fibroblast indicated strong signals and colocalization in the cytoplasm, confirming our earlier observation of the role of HABP1 in HA-mediated cellular signaling. However, in F-HABP07 cell line not only there was a reduced HA signaling probably generating HA oligomers; but also HABP1 did not colocalize with HA, even though the level of HABP1 is high in this cell line. Thus, micrograph of cytochemical staining for HA clearly exhibited the depolymerization and significant reduction in level of HA in F-HABP07 cells as compared to parental control cells at 60 h of growth (Figure **7A**). This observation was further validated by studying the reduction and depolymerization of HA in F-HABP07 cells using the fractionation of polymeric HA and the low molecular weight degraded HA oligosaccharides by polyacrylamide gradient gel electrophoresis. We verified the status of polymeric HA in both the cell lines F111 and F-HABP07. HA enriched fractions purified from the two cell lines grown for 60 h were either digested with bovine testicular hyaluronidase (BTH) or kept untreated. Subsequently, both undigested and BTH digested products were run in a 5-20% polyacrylamide gradient gel as per the procedure described in ‘Materials and Methods’ in order to separate the various fragments of HA. The polymeric HA remained on the upper end of the gel whereas; BTH degraded HA migrated to the other end. Using purified HA from the two cell lines, a significantly decreased polymeric HA level in F-HABP07 compared to F111 was clearly evident in the upper bands of the silver nitrate stained gel. More oligomeric fractions were found in the F-HABP07 than polymeric HA. The polymeric HA further disappeared upon subsequent treatment with BTH (Figure **7B**). The expression level of HAS2 was found to be almost similar in both F111 and F-HABP07 upon immunoblotting (Figure **7C**); suggesting that reduction in HA is not at the level of synthesis, but due to degradation. 

### Reversal of ROS induced autophagic vacuole formation by supplementation of polymeric HA, an endogenous ROS scavenger

**Figure 8:**
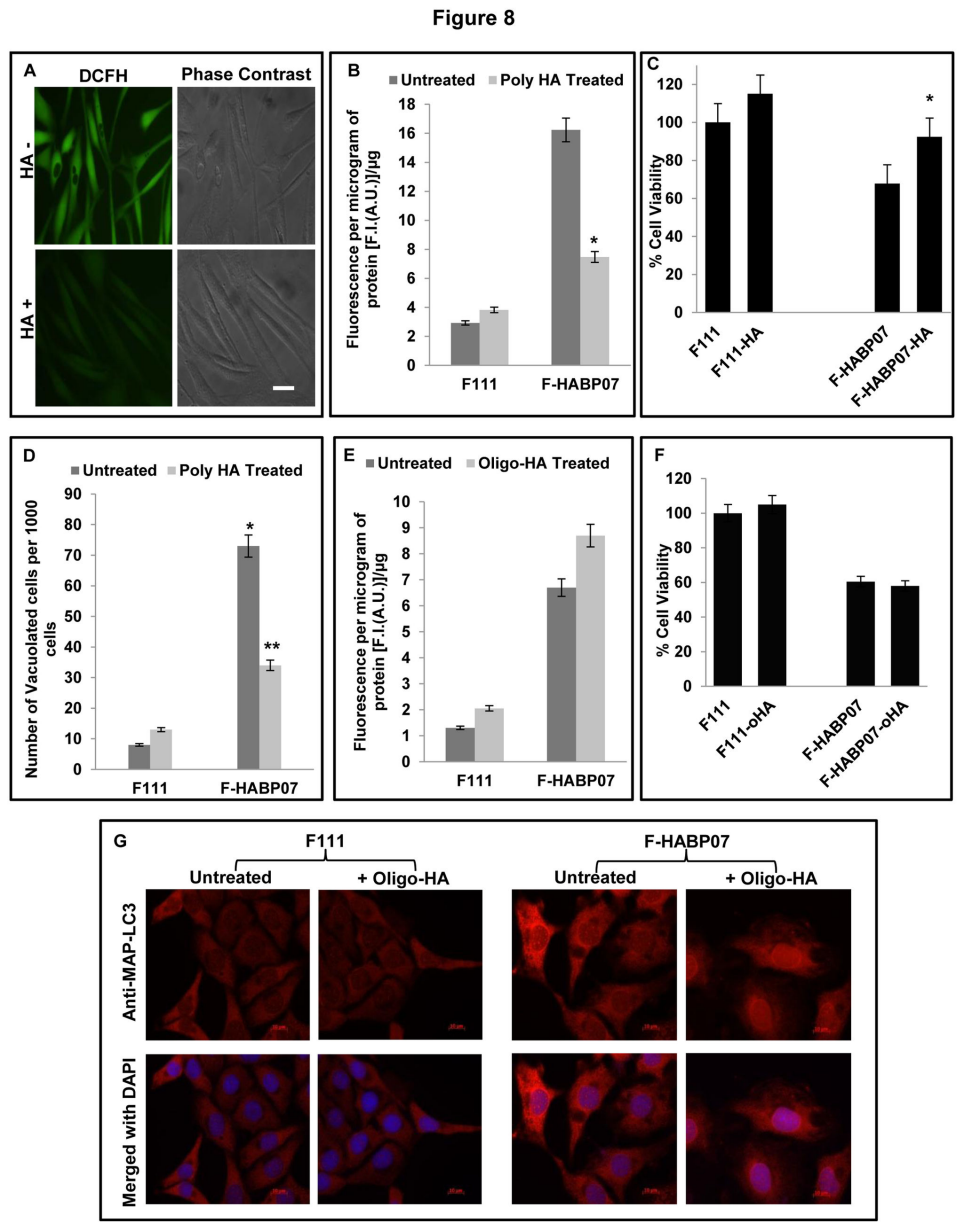
Reversal of ROS induced autophagic vacuole formation in F-HABP07 upon polymeric HA supplementation. [**A to D**] ***PolymericHAsupplementationrestorescellularhomeostasisinF-HABP07cells***-- As a result of significant reduction in ROS generation as evident from the decreased fluorescence intensity upon H_2_DCFDA treatment (**A** and **B**); about 25% revival of growth was observed in F-HABP07 cells (**C**). Single factor one-way ANOVA with difference at a level of p ≤ 0.05 between groups considered to be statistically significant indicates the decrease in ROS level and increased survivability in F-HABP07 cells as significant, which is denoted in the figures with * (**B** and **C**). Reduction in number of vacuolated cells was also detected upon polymeric HA supplementation. MDC, specific stain for autophagic vacuoles used to detect vacuolated cells in the control untreated and HA supplemented cells grown for 60 h. The differences were scanned and counted under the fluorescence microscopic fields. At least from 1000 cells, the total number of vacuolated cells was counted. After repetition of all experimental sets at least three times, the cumulative tabulated data were presented in the bar diagram showed the significant down regulation of autophagic vacuoles in F-HABP07 grown in HA supplemented medium. Asterisks represent level of significance as * denotes p<0.001 for F-HABP07 compared with the control normal fibroblast and ** denotes p<0.001 for F-HABP07 grown in media supplemented with polymeric HA compared to untreated F-HABP07 cells. A significant reduction in the number of vacuoles in F-HABP07 was observed at 60 h upon polymeric HA supplementation (150µg/ml) at 36 h (**D**). Thus, suggesting a role of HA in scavenging ROS. [**E to G**] ***OligomericHAsupplementationwasineffectiveinrestorationofcellularhomeostasis***-- Since, supplementation of polymeric HA caused a revival of cellular homeostasis, the effect of o-HA on F111 and F-HABP07 cells were also checked upon supplementation. ROS assay performed using H_2_DCFDA showed negligible fold change in both the cell lines (E), while MTT assay revealed no effect on survivability of F111 and F-HABP07 cells after supplementation of o-HA (**F**). Moreover, o-HA supplementation was found to have no effect on the expression pattern of the autophagic marker, MAP-LC3 in the normal fibroblasts, F111 (**G**); since both the treated and untreated cells showed a similar immunostaining for MAP-LC3.

To examine whether the known endogenous ROS scavenger HA has any rejuvenating effect on the cellular homeostasis of F-HABP07, these cells along with the control normal fibroblasts were supplemented exogenously with polymeric HA (150 μg/ml) in the culture medium at 36 h. As shown in Figure **8A**, the fluorescence intensity of ROS sensitive dye H_2_DCFDA in F-HABP07, significantly reduced after HA supplementation. Dense elongated F-HABP07 cells with broader diameter were visualized under phase contrast after HA supplementation along with reduced fluorescence when compared with untreated controls (Figure **8A**). Moreover, the ROS level assayed fluorometrically after using H_2_DCFDA, showed significant quenching of ROS up to 8 folds in F-HABP07 cells after polymeric HA supplementation (Figure **8B**). Simultaneously, the growth rate of HA treated and untreated cells was assayed using MTT dye. According to earlier reports growth retardation was observed in untreated F-HABP07 cells as compared to F111 cells. Supplementation of polymeric HA significantly restored the growth of F-HABP07 cells by about 25% as evident from the cell viability curve (Figure **8C**). Reduction in ROS level after HA supplementation suggests a role of HA as a ROS scavenger. To correlate ROS level and the appearance of vacuoles with polymeric HA supplementation, reduction in number of vacuolated cells were discerned in F-HABP07 using the autophagic marker, the fluorescent dye MDC. Polymeric HA supplementation resulted in the significant reduction of vacuolated cells by about 50 % in F-HABP07 cells; while the parental cell line showed no significant change (Figure **8D**). However, if supplementation was given at a later time, there was insignificant change in the number of vacuoles (unpublished observation). 

Contrarily, o-HA supplementation similar to polymeric HA supplementation showed negligible fold change in ROS (Figure **8E**) and cell growth in F111 and F-HABP07 cells (Figure **8F**). Immunostaining with anti-MAP-LC3 for the autophagic marker indicated that the expression of the protein remains unchanged in F111 cells after o-HA treatment. However, the upregulated MAP-LC3 in F-HABP07 cells not only remains upregulated but also gets translocated to nucleus upon o-HA supplementation (Figure **8G**). 

These observations imply that external supplementation of o-HA at 36 h does not affect the redox and autophagic status of F111 which has high level of polymeric HA. Conclusively, our overall findings also suggest that ROS induced autophagic vacuoles in F-HABP07 cells can be reverted by the supplementation of ROS scavenger or antioxidant, polymeric HA.

## Discussion

Autophagy, primarily a catabolic process, involving the regulated turnover and elimination of proteins and cellular organelles, promotes survival under metabolic stress, but it can also be a mediator of cell death if executed to completion [42]. Although not much is known about its precise regulation, the ability of a large number of stimuli to trigger autophagy suggests the implication of numerous signaling pathways responsible for the formation of autophagosomes. Autophagy has been supposed to be modulated by mainly two regulatory proteins; firstly mammalian target of rapamycin (mTOR), a cell growth regulator integrating growth factor and nutrient signals and secondly AMP activated protein kinase (AMPK), a key energy sensor regulating metabolism. AMPK promotes autophagy through inactivation of the nutrient sensitive mTOR complex 1 (mTORC1) [43,44]. AMPK mainly considered as a pro-survival kinase might lead to cell death upon sustained activation [44] and one of the factors implicated in its activation is oxidant stress or ROS [45]. Using a stable fibroblast cell line overexpressing HABP1, which generates excess ROS and subsequently undergoes apoptosis; we confirmed ROS-mediated regulation of autophagy prior to apoptosis as evident from the following observations. Increased autophagic vacuoles were observed in F-HABP07 cells during oxidative stress as revealed from MDC staining. Period of autophagic vacuolation demonstrated upregulation of autophagic markers Beclin 1, MAP-LC3-II and the autophagic modulator, the lysosomal protein DRAM. Vacuole formation in F-HABP07 was reduced by 3-MA, the known inhibitor of autophagy. Inhibition of lysosomal proteolysis by Baf A1 confirmed increased autophagic flux taking place upon HABP1 overexpression in normal fibroblasts. Proteasomal inhibitor induced vacuole formation was observed in F111 and vector transformed fibroblasts, whereas F-HABP07 underwent cell death, perhaps due to extreme stress. At 60 h of cell growth the expression of tumor suppressors PTEN and p53 were elevated in F-HABP07 cells coinciding with apoptosis induction. Excess ROS generation in F-HABP07 was instrumental in the appearance of autophagic vacuoles and reduced level of the endogenous ROS scavenger, HA. This can be corroborated with the observation of decline in autophagic vacuole formation due to diminished ROS level after external supplementation of polymeric HA. 

Autophagy, the evolutionarily conserved stress response system, was evident in HABP1 transformed fibroblast cells (F-HABP07) when the cells were undergoing oxidative stress, but the oxidant level showed a steep rise from 48 h to 60 h of growth (about 6 to 12 fold) [16]. Subsequent to administration of 3-MA, a potent PI3-kinase inhibitor, inhibition of autophagic vacuolation in this cell line was only possible in the early period of growth (32 h). This observation suggested that once a high level of ROS (6 to12 fold) is attained, the process of autophagy cannot be attenuated. 

Beclin 1 is a component of the nucleation complex that along with class III PI3K/p150 forms a part of the initial stage of the process of autophagy; which might be the reason for its higher expression at 36 h in F-HABP07 cells. While, expression of MAP-LC3-II, a part of the autophagic membrane complex, was highest at 48 h of growth; corroborating with the observation of the maximum number of vacuoles at that stage. The expression of Beclin 1 decreased to basal level at 48 h, as autophagic vacuoles were already formed by that time. But, since the cells showed a surge in ROS from 48 h to 60 h of growth, they tried to adapt to that oxidative stress that is evident from the increased expression of Beclin 1. From the observations it was apparent that the cells were unable to overcome the stress and a fall in mitochondrial membrane potential (Δψ_m_) was witnessed at 60 h of growth along with apoptosome complex formation. As reported earlier, the cells were found to undergo apoptosis at 72 h of growth. Moreover, mitochondrial accumulation of HABP1/p32/gC1qR has been reported to inhibit respiratory chain complex I activity [16]. Thus, it is conceivable that the very same cell line exhibits excess ROS generation, autophagy and apoptosis. ROS generated as a by-product in the mitochondrial electron transport chain can act as signaling molecules to regulate pathways leading to both cell survival and cell death. Autophagic vacuolation has been reported to act as a cell survival strategy during starvation-induced oxidative stress [25,26]. Our observations relating to the inhibition of ETC complex I and autophagy corroborates with a recent report [27] where inhibition of the mitochondrial electron transport chain (mETC) by inhibitors rotenone and TTFA blocked Complex I and Complex II respectively resulting in autophagic cell death of HEK 293, cancerous cell lines U87 and HeLa cell lines by the generation of ROS. Additionally, the induction of both autophagy and apoptosis has also been reported in the human Glioma U251 cells upon oxidative stress [46]. Autophagy and apoptosis may be triggered by common upstream signals [47,48], and sometimes these result in combined autophagy and apoptosis. In other instances, the cell switches between the two responses in a mutually exclusive manner [49]. 

Our observation on role of HABP1 in autophagic regulation can be supported by the previous report indicating the physical interaction of HABP1/ p32 with both murine and human smARF, an autophagy inducer. HABP1/ p32 co-localizes to the mitochondria with these short isoforms, stabilizes the mitochondrial smARF which otherwise is short-lived and undergoes degradation by proteasome-mediated process. Knocking down p32 protein by p32 siRNA significantly reduces the steady state levels of smARF by increasing its turnover and thus reduction in autophagy and mitochondrial membrane dissipation [33,34]. Although this protein is ubiquitous in nature, it is not expressed equally throughout the cells. It shows differential localization in different cells lines under various physiological conditions leading to multivariate binding of ligands and thus explaining its multifunctionality. Mitochondrial localization of HABP1 in F-HABP07 has already been reported from our laboratory [16]. Recent report suggests the involvement of p32/HABP1 in autophagy for promoting degradation of parkin, a neuroprotective protein functioning as E3 ligase in ubiquitin proteasome system (UPS) [50]. Parkin targets several substrates for ubiquitination and defective mitochondria for autophagy. Thus, p32 has been proposed to regulate mitochondrial morphology and dynamics by amending parkin protein level via autophagy [50]. Out of the two major degradative pathways present in a cellular system, the UPS is majorly involved in the degradation of short-lived proteins while the conserved autophagic system is involved with the elimination and recycling of worn out long lived proteins and organelles. Suppression of the UPS leads to the build-up of misfolded proteins in the ER, which may cause significant ER stress. It has been demonstrated that autophagic degradative machinery responds and compensates for the inhibition of the proteasomal system to regulate ER stress and cell viability [51]. Our study indicated that MG132 treatment proved fatal for F-HABP07 cells that were pre-stressed due to excess generation of ROS leading to autophagic manifestation and could not sustain subsequent stress from the inhibition of proteasomal degradation.

Our observation on upregulation and nuclear translocation of the tumor suppressor PTEN, during 60 h of growth in F-HABP07 also coincided with a huge surge in ROS generation from 48 to 60 h and induction of apoptosis [16]. PTEN is implicated in autophagy by the inhibition of Akt/PKB signaling mediated by its lipid phosphatase activity [29]. It is reported to be constitutively active during elevated ROS levels and is a crucial and common mediator in ROS generation and neuronal cell death [52]. Also, there are several reports linking overexpression or induced expression of PTEN with cell cycle arrest and induction of apoptosis in several types of cells, like MCF-7, Jurkat T cells, fibroblasts etc. [53-55]. Also, the data showing increased expression of p53 at 60 h appears to support the ROS-mediated apoptosis, implying ROS as an upstream signal triggering p53 activation and a downstream factor that regulate apoptosis [56]. A microarray analysis of H_2_O_2_-treated human cells identified one-third of the 48 highly H_2_O_2_- responsive genes as targets of p53 [57]. At basal or physical levels, p53 has a subtle but vital function in maintaining ROS at non-toxic levels through transactivation of several antioxidant genes. On the contrary, overexpression of p53 transactivates a series of p53-induced genes (PIGs) and many of these *PIG*s encode redox-active proteins including two ROS-generating enzymes, NQO1 (quinoneoxidoreductase, PIG3) and proline oxidase (POX, PIG6). Upregulation of these pro-oxidant enzymes leads to oxidative stress and consequently to apoptosis [56,58]. Apart from the role of p53 in apoptosis, it has also been incriminated to have a function in autophagy. The p53 activation is implicated in the repression of mTOR leading to autophagy [59]. Also p53 can transactivate target genes like DRAM and PTEN which are believed to have a crucial role in the regulation of autophagy [60,61]. Overexpression of DRAM leads to accumulation of autophagic vacuoles, while it’s silencing is associated with downregulation of both autophagy and apoptosis [60,62,63]. Thus, increased expression of p53 and DRAM seem to play an important role in the appearance of autophagic vacuoles and induction of apoptosis in the HABP1 transformed fibroblasts, F-HABP07. 

Excess ROS is also known for its degradative action on HA polymer, generating HA oligosaccharides owing to its ROS scavenging property [64,65]. This could be instrumental in substantial reduction in polymeric HA level observed in the HABP1 overexpressing fibroblast generating excess ROS. This conceivably is reflected in significant reduction in the number of vacuoles in F-HABP07 observed at 60 h upon polymeric HA supplementation (150µg/ml) exogenously at 36 h. Reduction in level of ROS generation was evident in the HA supplemented F-HABP07 cells, from the decreased fluorescence intensity upon H_2_DCFDA treatment. But, supplementation of HA at a later stage did not show a similar outcome (unpublished observation). The induction of apoptosis after 60 h may be related to the ROS-induced depolymerization of HA since; HA oligosaccharides are reported to be involved in signaling cascades inhibiting anchorage-independent growth of several cell types and induction of apoptosis [9]. It has also been suggested that HA oligomers stimulate the expression of PTEN [66-68], a phosphatase that suppresses the PI3-kinase/AKT cell survival pathway leading to cell growth inhibition and apoptosis. Earlier report from our laboratory has revealed that supplementation of known antioxidants like pyrrolidinedithiocarbamate (PDTC), N-acetyl-cysteine (NAC), tiron and curcumin reduces oxidative stress in F-HABP07 and the cell line can serve as a tool to identify unknown antioxidants [69]. Co-localization studies between HA and HABP1 and mitotracker in F111 and F-HABP07 revealed differential interaction between HA-HABP1 in the two cell lines [16, supplementary data]. Oligomeric fragments of HA in F-HABP07 did not colocalize with the mitochondrially localized HABP1 leading to mitochondrial dysfunction [16], indicating a disruption of the normal physiological event of HA-HABP1 interaction which is otherwise occurring in normal fibroblasts. Additionally, our present data is supported by a recent report of the cytoprotective activity of HA on oxidatively stressed chondrocytes indicated by decreased mitochondrial DNA damage, enhanced DNA repair capacity, increased cell viability and reduced rate of apoptosis [70]. 

## Conclusion

We report that overexpression of HABP1 in normal fibroblasts induces autophagy with the upregulation of autophagic markers and modulators in a ROS-dependent pathway that depolymerizes the endogenous antioxidant HA. This autophagic vacuolation could be inhibited only in the early redox stage either by inhibition of PI3 kinase activity or the use of antioxidants like polymeric hyaluronan.
